# Voltaire's *Candide*, medical students, and mentoring

**DOI:** 10.1186/1747-5341-2-13

**Published:** 2007-07-03

**Authors:** Thomas J Papadimos

**Affiliations:** 1 University of Toledo, College of Medicine, Department of Anesthesiology, Toledo, Ohio, USA

## Abstract

In Voltaire's work, *Candide*, a young, naïve man, who has been taught that humans live in the best of all possible worlds, is thrust into the world only to find that this may not be so. He learns over time to balance his optimism with the skepticism he acquires through experience. While today's medical students are not naïve like the character Candide, they, nonetheless, carry an impression of the ideal medical practice, along with the expectation of a successful medical practice. Good mentors and role models are important to students in order to temper their optimism, control their skepticism, and to help them to be realistic, not only about their expectations of medical practice, but what society expects of them.

## Background

*Candide *is a classic literary work that allows us to consider the parallel between literature and the education of medical students. Medical student mentors can learn much from its classic characters. By drawing on the humanities, mentors can enrich their perspectives and deepen their comprehension of human interactions, especially in regard to those who teach and those who are taught.

Voltaire's minor work, *Candide*, persists as one of his major contributions to literature and satire:

"It is a supremely wrought tragicomedy that slyly and irresistibly induces us to laugh at and simultaneously reflect upon the most dreadful events that befall humankind. It appeals to us today because, nearly 250 years after its publication, it has lost none of its relevance or satirical sting. It is particularly modern and pertinent because its dark comic vision is essentially in keeping with our own awareness of what separates our need for order, clarity, and rationality from the brutal reality of a chaotic world [1]."

While today's medical students are not naïve like Voltaire's character, Candide, the journey of medical students' *expectations *will be compared with that of Candide using Voltaire's wit and understanding by primarily borrowing his story's characters and venues to parallel the 21^st ^century medical students' environment.

The young, naïve protagonist, Candide, a hero who seeks to be happy against all odds and catastrophes, believes he lives in the best of all possible worlds. The freshness of his naivete is reflected in medical students who view the practice of medicine as the "best of all possible worlds." A seat secured in a medical school class is something that the student has worked hard to attain, and at this time of their early adulthood it is a most idealized adventure on which to embark.

However, society at large has dispatched much of the myth surrounding the idealized training of physicians. In John Updike's introduction to Samuel Shem's novel, *The House of God*, he applauds the author's initial attempt to relieve us of the illusion that,

" ...their training and expertise and saintly dedication have purged them of all the uncertainty, trepidation, and disgust that we would feel in their position, seeing what they see and being asked to cure it. Blood and vomit and pus do not revolt them; senility and dementia have no terrors; it does not alarm them to plunge into the slippery tangle of internal organs, or to handle the infected and contagious. For them, the flesh and its diseases have been abstracted, rendered coolly diagrammatic and quickly subject to infallible diagnosis and effective treatment [2]."

It is uncanny how Voltaire's *Candide *is applicable to the situation of medical students. So much so, that in his introduction to *The House of God *John Updike remarks that Shem's hero, Roy Basch (a new intern), "suggests Voltaire's *Candide *in his buoyant innocence and his persistent–for all the running hypochondria of his hectic confessional narrative–health [2]."

Most of  today's applicants to medical school remain idealistic, but are encumbered  by young adult developmental issues, incur great financial debt for their  education, and retain an element of naivete in their approach to their  chosen profession. It is not that they are naïve, but that they have a naivete of expectations in regard to their future medical practice. Their naivete of expectations will vanish with time and their optimism will be tempered with experience. Hopefully, skepticism will not overwhelm them, although it is natural, even protective at times. Support of the student by effective role models and mentors throughout their clinical years is of paramount importance to ensure their successful navigation of expectations and reality during their professional maturation.

## Cunégonde: the origins of the voyage

We can consider Candide's Westphalian paradise as the comfortable undergraduate situation of medical students where, like Candide, their voyage begins involuntarily when they graduate from college. Candide is not a college graduate, but his "voyage begins involuntarily when he is kicked out of his Westphalian paradise by the old Baron de Thunder-ten-tronckh..." for being affectionate with the Baron's daughter, Cunégonde [3]. Both Candide and the medical student leave their comfortable surroundings hoping to secure a dream. Candide wishes to be reunited with his true love, Cunégonde, whom he lost on his departure from Westphalia. For medical students, in the narrowest sense, their Cunégonde is the ideal practice of medicine. In a much larger sense, Cunégonde represents a more broad ideal regarding a protected life; an idyllic situation where all patients get well, all patients love their physicians, where there is remuneration for all patient encounters, where all patients can access healthcare, partake of the most advanced technology, and where malpractice is something that happens to someone else. However, the ideal of the "protected life" may only occur for medical students in their teaching environment. This environment should be a place where they can be nurtured and cared for before their inevitable confrontation with the practice of medicine. It is a place where good mentors can make a difference.

## Pangloss: the optimistic philosophy of the student's pre-morbid state

Voltaire wrote *Candide *as an objection to Gottfried Wilhelm Leibniz's theory that God created the universe as the best of all universes, or worlds. He mocked Leibniz using the character Pangloss, a philosopher who teaches an optimistic philosophy that is the antithesis of the world Voltaire creates around Candide, through violence and mayhem. Pangloss's "best of all worlds" view, though, is accepted by the naïve Candide [4].

Students do, in fact, demonstrate unrealistic optimism. McGee and Cairns demonstrated in a study of 257 medical students that were asked to document their belief about 17 different health problems, varying levels of unrealistic optimism about their beliefs and how these health risks affected them [5]. The "unrealism" related to optimism became a concept that was taught in class to give students "feedback on their own cognitive processes and illustrating that they have cognitive distortions similar to those of patients [5]."

The "Pangloss" of medical students is a crystallization of the medical students' pre-morbid state (before medical school). In other words, the "Pangloss" of medical students is their idealized expectations of medical practice, fueled by the perceptions of family, society, history, and the media, all of which perpetrate and perpetuate a medical/physician ideal. Additionally, this premorbid state may be further indurated by the tendency of medical schools to highlight their own glorious past, their research endeavors, and the medical field in general. Therefore, this "Pangloss" of medical students is their usher into the great hall of medicine where reality, armed with the crossbow of skepticism, awaits them.

## Martin: the skepticism of the experienced medical practitioner

Martin was an old philosopher that Candide befriended in Surinam. Candide chose him as a companion because he seemed to be "that person who appeared to him the most deserving of compassion and the most truly dissatisfied with his condition in life [4]." His selection as Candide's companion came about after a Dutch judge and a ship's captain both victimized Candide, leading to his conclusion that men were wicked [4]. He then made it known that he would provide room, board, and payment for the most unfortunate person in Surinam. Many applied, but Martin was selected. Martin was a scholar, an honest man who had been robbed by his spouse, assaulted by his son, persecuted by local clergy, and fired from his job of ten years. These experiences made him a skeptic. For example, Martin lamented that the had seen neighboring cities try to exterminate each other, family members try to hurt one another, that people were starving, and that the rich treated the poor harshly, to which Candide replied, "an yet there is some good in the world [4]." Martin countered, "Maybe so, but it has escaped my knowledge [4]." Amid adversity Martin cannot find buoyancy. A true mentor will find a teaching opportunity in an adverse situation; a lesson learned today is tomorrow's catastrophe averted. The character of Martin represents the skeptic, Pierre Bayle, one of Voltaire's favorite opponents of philosophical optimism [6].

In the context of this paper Martin represents the seasoned medical practitioner's view of medical practice, not jaundiced, but Bayle qua Martin, understanding as Voltaire did that the world is both good and evil. There are good days and bad days in the practice of medicine. Some patients survive horrible illnesses and some do not. Sometimes patients and insurers pay for services rendered, sometimes they do not. On rare occasions a physician will be sued for malpractice, and on occasions he or she will lose, "You are very hard in your beliefs," said Candide. "It is because," said Martin, "I have seen the world [4]."

Physicians' skepticism starts in medical school. Griffith and Wilson confirmed changes in student attitudes toward patients in their third year of training, especially for those who are elderly or in chronic pain [7]. Students were of the opinion that most of the elderly were demented and those with chronic pain were drug seekers. Furthermore, "students also became less idealistic toward the medical profession, believing fewer physicians love what they are doing, and having less faith in certain therapies [7]." According to Voltaire, on some days there are just too many obstacles to the idyllic life, such as Bulgarians, tempests, shipwrecks, and earthquakes abounding [4].

## Cacambo: support of the inexperienced

Cacambo is introduced in Chapter XIV as Candide arrives in Paraguay:

"Candide had brought with him from Cadiz such a footman as one often meets on the coast of Spain and in the Colonies. He was one-quarter Spanish, the son of a half-breed and was born in Tucuman. He had been a singing boy, sexton, sailor, monk, peddler, soldier, and a lackey [4]."

He was wise and did his best to keep Candide on a safe path of good decision-making. In many ways his role was that of a teacher, in that he provided crucial support to Candide throughout his journey. He had many jobs in his lifetime, even that of a lackey. All faculty members remember being interns, or lackeys. Faculty members usually have a history of varied jobs or experiences, and in their quest to teach and role model they must take positions of support as the "wingmen" (military air support term) of medical students.

The only way to keep overwhelming skepticism at bay, that is skepticism that is neither natural nor protective, is for there to be role modeling and mentoring by those who teach the students. Where does Voltaire illustrate someone who inspires confidence, demonstrates moral uprightness, and is intelligent? While some may think Voltaire has no faith in humanity, one will only have to look to Cacambo:

"He knows both native American and European languages, and deals capably with both the Jesuits and Biglugs. He suffers fewer gross misfortunes than any other character, less out of luck than because of sharp wits, and he lives up to Candides's trust when Candide sends him to fetch Cunégonde. Any reader tempted to conclude that Voltaire has no faith in human nature must reconsider when faced with the example of Cacambo [8]."

Medical students need their mentors to be Cacambos: morally upright, confidant, intelligent, trustworthy, and sharp of wit to inculcate the necessary skills regarding the measurement of skepticism and optimism in their approach to the practice of medicine. Teachers must give them the information they need and mentor them through necessary experiences and situations. They must become Cacambos.

## Expectations of medical students: the El Dorado ideal

El Dorado was Voltaire's perfect world, a place where optimistic philosophy flourished, a land Candide visited with Cacambo. It was a place where honest people could be found and were contented with their lot in life, "I am very ignorant sir, but I am contented in my ignorance..." admitted an inhabitant to Candide [4]. It was a place where the ground was littered with "the pebbles and sand, which we call gold and precious stones [4]." It was a place where, upon visiting the king, "Twenty beautiful young virgins in waiting welcomed Candide and Cacambo as they stepped from the coach, led them to the bath, and dressed them in robes made of the down of humming birds... [4]." El Dorado was a utopian welfare state. Everybody enjoyed everything and everyone's needs were met. It was a place Voltaire felt could never exist.

Nonetheless there is an "El Dorado" of medical practice in the medical students' mind, the ultimate destination of their voyage in medicine. While he or she may know there are pitfalls along the road, they hope for idealized conditions such as insured patients, honest administrators, prompt-paying insurers, a few "precious pebbles" that they may acquire along the way, and hopefully, an understanding spouse or partner.

As the medical student approaches his or her senior year the optimism remains for a bright future. Although they are encumbered by a huge debt for their medical education their quest for the El Dorado of medicine remains their ideal [9]. However, while the Pangloss of their optimism whispers in one ear, the Martin of skepticism has begun to whisper in the other. Indeed, both optimism and skepticism have their place, but medical students need, as Candide did, someone to support them in this journey.

## Expectations of society: the community ideal

Advanced medical skills, paying patients, and a few "precious pebbles" are hopeful expectations of medical students. But does society have expectations of physicians?

While in Paris with Martin and an Abbe, Candide came across a particular situation (the burial of royalty), and the reaction of the local populace surprised him:

"Lord," said Martin. "What do you expect? It is the way of these people. Imagine all the contraindications, all the inconsistencies possible, and you may meet with them in the government, the courts of justice, and the public spectacles of this odd nation." "Is it true," asked Candide, "that the people of Paris are always laughing?" "Yes," replied the Abbe, "but with anger in their hearts [4]."

So can it be with society's expectations of physicians. Society may be wearing an angry smile. The angry smile may not only be that of a patient, but also that of managed care, private insurance companies, or the government. The government may play a particularly pivotal role because it represents large social trends and ambivalences. The community wants to be able to trust physicians and have confidence that they will do the right thing. The community does not like what has happened to the doctor-patient relationship. This relationship has been transformed "by substituting questions of cost and benefit for traditional relations of care and trust...the stronger interests have prevailed, with the public reduced to bystander status and medicine itself more passive than active" [10]. It is important mentors make sure that students understand that:

"Medicine depends on more than competence and expertise, essential as they are. It cannot function as an institution without good faith on the part of the provider, patient and public as a whole. The root of the public's trust is the confidence that physicians will put the patients' welfare ahead of all other considerations, even the patients' momentary wishes or the physicians' monetary gain. It is the function of medicine as a profession to safeguard and promote this trust in the society at large. This point could be phrased by the maxim: 'Medicine must always be treated as a public good, never as a commodity [10]."'

## Cultivating our garden: a coalescing of ideals

In the conclusion of Voltaire's tale Candide, Pangloss, Martin, Cunégonde, and Cacambo have settled down on a small farm outside of Constantinople. One day while returning to the farm Candide, Pangloss, and Martin (Cacambo had not accompanied them on this particular outing) passed a "good-looking old man, who was enjoying some fresh air at his doorway under the alcove formed by the boughs of orange trees [4]." The old man invited them in. Candide assumed the old Turk had a vast estate, but he did not, "I have no more than twenty acres of ground, the whole of which I cultivate myself with the help of my children, and our labour keeps us from three great evils–boredom, vice and want [4]."

Candide reflected upon the old Turk's conversation, as did Pangloss and Martin. Candide commented to Pangloss and Martin "that we must cultivate our garden [4]." Pangloss agreed and stated that men should not be idle, to which Martin added, that men should work together without disputes. Thereafter all the inhabitants of the farm pooled their talents in a community effort that allowed the enterprise to produce good harvests, and these good harvests (economic and social) were possible because Candide had been guided by the role and active presence of his "good mentor", Cacambo.

The faculty members of medical schools, as role models and mentors, must be involved in an enterprise that produces good harvests. Collective efforts must be directed at producing, not only competent practitioners, but physicians who know what to expect and what is expected of them. For this to happen, able mentors are required. Able mentors are teachers with integrity, experience, and flexibility [11]. The best mentors "do not promote their own agendas, use their mentees as free labor, take credit for their mentees accomplishments, and do not try to create a clone who mimics the mentor's career path, philosophy, and opinions [11]."

Voltaire's *Candide *is a clever, superficial, and sarcastic work. However, this commentary was not meant to paint a cartoonish comparison between Candide and today's medical student. Any caricature of medical students' plights regarding expectations is unintentional. My concern is primarily directed at the faculty's role as mentors. As teachers, academic physicians play the roles of Pangloss, Martin, and Cacambo interchangeably. They bring optimism, skepticism, and support to the teaching of the healing arts. They teach medical students to hone their skills, temper their optimism, control their skepticism, and be realistic about their expectations of medical practice. Students have expectations of their medical practice, while mentors and society have expectations of the students' *practice of medicine*. It is not that a medical student's El Dorado is not attainable, it is. Through role modeling and mentoring, teachers of medicine can help students realize the true El Dorado of medicine; a state where optimism and skepticism are not only balanced in an effort of acquisition, but also in an effort of proffering the art of healing to the community. A balance Voltaire hoped that we would strike; in a garden that he'd hoped we would cultivate.

In the course of a medical education, and on into medical practice, a student will carry the optimism of Pangloss and the skepticism of Martin, balancing them delicately throughout. The experiences of students and their resultant decisions, influenced by able mentors, will affect and complete their professional soul; to which Candide would respond, "Excellently observed, but we must cultivate our garden [4]." However, the cultivation of this garden requires a lifetime of effort.

## Conclusion

Voltaire wrote this "minor" work as an objection to the philosophical optimism of Leibniz. He presents us with an optimistic youth who must deal with waning optimism and overwhelming skepticism in a world gone wrong, a world of declining expectations. His only effective guide was Cacambo, a balanced and experienced individual...a mentor. We need professionalism in role modeling, teachers of excellence, "who excite admiration and emulation," and then become mentors of the highest caliber [12]. Such mentors must foster optimism with a balanced skepticism in medical students, who, in turn, have great expectations of a society that has great expectations of them.

## Competing interests

The author(s) declare that they have no competing interests.

## Authors' contributions

TJP was responsible for the entire manuscript.
